# Short-Window Micro-Behavioral Triage for High-Throughput Firewall Telemetry: Limits, Features, and Operational Feasibility

**DOI:** 10.3390/s26144600

**Published:** 2026-07-20

**Authors:** Kadir Kesgin, Vedat Tümen, Erdal Akın

**Affiliations:** 1 Department of Computer Technologies, Gönen Vocational School, Bandırma Onyedi Eylül University, 10250 Bandırma, Türkiye; 2 Computer Engineering Department, Bitlis Eren University, 13100 Bitlis, Türkiye; vtumen@beu.edu.tr; 3 Department of Computer Science and Media Technology, Malmö University, 205 06 Malmö, Sweden; 4 Sustainable Digitalisation Research Centre, Malmö University, 205 06 Malmö, Sweden; 5 Biofilms Research Center for Biointerfaces (BRCB), Malmö University, 205 06 Malmö, Sweden

**Keywords:** network telemetry, firewall logs, high-throughput monitoring, dense traffic slices, short-window analytics, anomaly detection, robust scaling, Shannon entropy, micro-behavioral profiling, real-time feasibility, IoT security, edge monitoring, network sensing, privacy-preserving telemetry

## Abstract

High-throughput firewall telemetry increasingly demands real-time interpretation from short, dense observation windows rather than long historical baselines. We operationalize *behavioral slicing*, a privacy-preserving approach that extracts interpretable micro-behavioral signals—timing irregularities, destination dispersion (entropy), and intensity cues—from sub-minute traffic segments. This study does not claim confirmed intrusion detection. Instead, it evaluates whether short-window firewall telemetry can support privacy-preserving candidate triage under high-throughput conditions and quantifies the limits, feature stability, detector complementarity, and real-time feasibility of such a pipeline. Our case study uses a *real* operational firewall telemetry slice that was irreversibly anonymized prior to analysis. Using unsupervised outlier detection with explainable summaries, the slice-level pipeline produces investigation-oriented anomaly candidates for triage, without making longitudinal routine or habit claims. Synthetic anomaly injection is used only for relative validation, while behavioral findings are derived from the real telemetry slice. Scenario-level ablations show strong separation for scanning and exfiltration but weak performance for low-variance C2 beaconing, indicating that behavioral slicing should be treated as a triage pre-filter rather than a universal detector. In the context of IoT and edge-network monitoring, firewall telemetry can be treated as a high-rate network sensing stream that reflects device-level communication behavior under operational constraints. Overall, results support behavioral slicing as an operationally feasible pre-filtering and triage method under extreme network load, with clear trade-offs and limits.

## 1. Introduction

Modern networks increasingly rely on high-throughput telemetry (e.g., flow records, firewall logs, and network events) to provide fine-grained, low-latency visibility for monitoring and security. At data-center, enterprise, and IoT-edge scale, telemetry systems must operate under tight resource constraints while sustaining massive event rates, motivating streaming architectures and compact measurement techniques [1,2,3]. In IoT and edge settings, recent *Sensors* studies similarly emphasize resource-constrained security architectures, multi-access edge computing for intrusion detection, lightweight models, and feature-extraction strategies for monitoring device-level network behavior [4,5,6,7,8,9]. A key scientific challenge follows: **how to extract interpretable, actionable signals from short, dense observation windows** when latency budgets prohibit long-horizon aggregation and when the available window is too short to justify routine or periodicity claims.

While User and Entity Behavior Analytics (UEBA) is often framed as a longitudinal problem that learns stable baselines from heterogeneous telemetry, operational monitoring frequently demands *real-time* decisions under high ingestion rates and imperfect attribution (e.g., missing or unreliable user identifiers) [10,11,12]. This motivates a complementary perspective: instead of requiring long history, treating dense, sub-minute windows as first-class analytic units for short-horizon behavioral inference in high-rate telemetry streams.

To address this gap, we operationalize *behavioral slicing* as a dense-slice analytics approach that extracts micro-behavioral signatures that remain meaningful at short horizons. Rather than attempting to infer long-term habits, we focus on interpretable micro-signals that can be computed and inspected in seconds, including timing irregularities (inter-arrival dynamics), destination dispersion captured via entropy, and intensity cues. This choice is consistent with prior anomaly research showing that short-term observations can be discriminative when features summarize distributional shifts and temporal dynamics. Information-theoretic measures (e.g., entropy over destinations/services) can localize acute changes without requiring long historical baselines [13,14,15,16,17], and inter-arrival patterns have long been used to characterize bursty behavior and irregular events in network telemetry [16].

Dense slices are also sensitive to artifacts common in operational logs: duplicates, counter-like fields, and heavy-tailed byte/packet distributions can destabilize classical normalization and z-score-based heuristics. Robust statistics provides principled tools for scoring under contamination; median/MAD-based estimators offer high-breakdown robustness under outliers [18,19]. Consistent with log-analysis surveys emphasizing the impact of preprocessing choices on validity and reproducibility, our pipeline explicitly incorporates deduplication and robust scaling/scoring to stabilize short-window analytics [20].

We analyze a 10 min operational firewall telemetry capture and report both the original dense 102 s high-load segment (with a peak rate of **1412 events per second (EPS)**) and a multi-slice stability analysis over consecutive short windows. We treat this dataset as a *real* operational telemetry slice (irreversibly anonymized prior to analysis) and evaluate micro-behavioral signals that remain meaningful in short horizons: inter-arrival time (IAT) dynamics, destination diversity/entropy, and application switching rates. When user identity is unavailable, we adopt an *identity-fallback* strategy by profiling entities at the IP level to preserve analytical coverage.

### 1.1. Hypotheses

Before presenting the empirical results, we state the four hypotheses used to organize the analysis:

**H1.**  *Anomaly candidates exhibit higher short-window burstiness than normal events.*

**H2.**  *Anomaly candidates exhibit higher destination-dispersion signals, especially destination entropy.*

**H3.**  *Isolation Forest and LOF expose complementary candidate sets because their global-isolation and local-density inductive biases respond to different short-window geometries.*

**H4.**  *Robust preprocessing, especially deduplication and median/MAD-based scaling, improves scientific fidelity under duplicate-heavy and heavy-tailed telemetry.*

These hypotheses are evaluated in the Results and interpreted in the Discussion; they are not introduced post hoc as confirmed claims.

### 1.2. Contributions

Our contributions are as follows:

1. We formalize **behavioral slicing** as a dense-slice analytics paradigm for high-throughput network telemetry, explicitly avoiding longitudinal routine or habit claims.
2. We define a compact, interpretable set of **micro-behavioral features** (IAT dynamics, destination entropy/diversity, and application switching) grounded in short-window and information-theoretic anomaly research [16,17].
3. We demonstrate that **robust preprocessing** (deduplication and median/MAD-based scaling) improves stability for short-window analytics in noisy, heavy-tailed telemetry streams [18,20].
4. We quantify **real-time feasibility** via end-to-end throughput benchmarking, showing substantial compute headroom relative to the observed peak load.

By reframing the problem as *dense-slice telemetry analytics* rather than long-horizon behavior modeling, we preserve scientific validity while targeting an operationally relevant regime: extracting interpretable, low-latency micro-behavioral signals from short, high-rate firewall telemetry segments.

## 2. Related Work and Literature Review

Our study lies at the intersection of (i) high-throughput network telemetry and measurement under resource constraints, (ii) short-window anomaly signals using interpretable dispersion and temporal features, and (iii) robust log/telemetry analytics in the presence of duplicates and heavy-tailed distributions. This positioning builds on broader anomaly-detection surveys and network-security critiques that distinguish statistical outlier discovery from operational intrusion claims [21,22,23,24]. We position *dense-slice analytics* (“behavioral slicing”) relative to these lines and explain why a short, high-rate window can still support scientifically meaningful, *short-horizon* claims without implying longitudinal routines.

### 2.1. High-Throughput Network Telemetry and Real-Time Constraints

Modern networks require telemetry systems capable of fine-grained, low-latency visibility while operating under tight compute and memory budgets.

Landau-Feibish et al. explicitly synthesize compact data structures for network telemetry in an ACM Computing Surveys article [3]; together with related streaming telemetry studies, the work shows how line-rate telemetry trades off accuracy, memory, and processing overhead [1,2].

This body of work motivates our setting: under high ingestion rates, the scientific objective is often not long-horizon baselining but extracting timely signals from short windows with minimal latency. Our notion of a *dense slice* aligns with this operational reality by treating a short, high-rate segment as a first-class analysis unit. For sensor-oriented IoT deployment, this framing also connects firewall logs to network sensing: edge gateways and security appliances observe communication traces from heterogeneous devices and can expose short-horizon behavioral evidence without requiring device payload inspection [4,5,9].

### 2.2. Short-Window Anomaly Signals: Entropy and Temporal Dynamics

When observation windows are short, features must remain informative without requiring long historical baselines. Information-theoretic measures (e.g., entropy of destination or service distributions) have a well-established history in network anomaly detection, capturing abrupt distributional shifts from focused communication to dispersed/scanning-like behavior [13,14,15,16]. These approaches are attractive for dense-slice analytics because they summarize distribution change compactly and interpretably, a property repeatedly emphasized in surveys of network anomaly detection and general anomaly detection [21,22,23].

Temporal dynamics are similarly important in short horizons: burstiness and inter-arrival time (IAT) irregularity can change rapidly under high-load conditions, while operational deployment must remain cautious about label scarcity, base-rate effects, and evaluation realism [24,25]. Accordingly, we center micro-behavioral profiling on destination entropy/diversity and IAT dynamics, complemented by application switching as a proxy for rapid context changes within the slice.

Beaconing-specific C2 detectors typically target cadence regularity, periodicity, or repeated low-variance communication over longer observation horizons. Our dense-slice setting is different: it uses a short, high-rate firewall segment to produce triage candidates rather than a cadence-specialized beaconing verdict. This distinction is important because the synthetic C2 beaconing stress test below intentionally represents a low-variance repeated-destination pattern that can be geometrically close to benign keep-alives and logging traffic in short windows.

### 2.3. Robust Preprocessing and Contamination-Aware Telemetry Analytics

Operational telemetry and logs are frequently affected by duplicates, counter-like fields, and heavy-tailed byte/packet distributions. A survey of deep-learning-based log anomaly detection highlights that preprocessing choices (parsing/encoding, normalization, artifact handling) materially affect detection outcomes and reproducibility [20]. A critical review of common log datasets further shows that benchmark assumptions can be misleading and underscores the need for transparent preprocessing and carefully scoped methodological claims [25]. These findings reinforce our design choice: deduplication reduces repeated artifacts, and robust scaling stabilizes feature distributions in dense slices where a small number of extreme events can dominate classical statistics [18].

Recent work also emphasizes that variable parts of structured logs (e.g., identifiers such as IPs or user tokens) can carry substantial anomaly signal. For example, the Variable Type Detector (VTD) analyzes statistical properties of variable fields to detect anomalies in strongly structured logs [26]. In our setting, this motivates the explicit handling of missing identity attributes and careful feature construction on the available identifiers.

### 2.4. Unsupervised Detectors and Inductive Bias in Dense Slices

Classic unsupervised detectors remain widely used due to simplicity and strong empirical performance. Isolation Forest isolates anomalies via random partitioning [27], while Local Outlier Factor (LOF) scores outliers via local-density deviation [28]. In short, dense windows, heavy tails and neighborhood geometry can cause detectors with different inductive biases to surface different candidate subsets. This motivates reporting detector diversity and agreement statistics, and it supports interpreting outputs as *model-dependent investigation candidates* in the absence of labels [6,7,8,20,25].

### 2.5. Behavior Analytics and Attribution Constraints (UEBA as Motivation)

Behavior-centric security analytics (including UEBA) is often framed as learning longitudinal baselines of users and entities from heterogeneous telemetry. In practice, however, identity attribution can be missing or unreliable, and real-time decisions may be required under high ingestion rates. Representative UEBA workflows highlight reliance on historical context and identity fidelity [10], and recent studies explore scalability and algorithmic diversity in enterprise settings [12]. At the same time, realistic evaluation remains challenging because suitable real-world behavior datasets are scarce and synthetic datasets may not reflect the instability of genuine behavior [11]. These constraints motivate our identity-fallback strategy: when user is missing, entity-centric profiling can be performed at the endpoint/IP level to preserve coverage within dense slices.

Table 1 and Table 2 provide a compact, non-duplicative summary of the contrast between longitudinal UEBA and dense-slice telemetry analytics, covering context, features, robustness, and modeling assumptions.

Table 1 and Table 2 summarize how dense-slice telemetry analytics differs from long-horizon baselining approaches (e.g., UEBA-style profiling): we operate under high-throughput telemetry constraints [3], tolerate incomplete attribution fields, and prioritize robust micro-signals (e.g., entropy and inter-arrival dynamics) that remain informative in short horizons [14,15,18]. This perspective also motivates explicitly reporting detector diversity (Isolation Forest vs. LOF) rather than assuming model agreement in unlabeled dense slices, since different inductive biases can surface complementary candidate subsets under heavy-tailed and bursty regimes [20,25,27,28].

## 3. Methods: Robust Micro-Behavioral Profiling

This section describes the end-to-end pipeline used to construct a high-throughput *behavioral-slice* representation, extract micro-behavioral features, and flag anomaly candidates for triage. Our design explicitly targets short, dense windows where classical longitudinal baselining is not defensible, and therefore emphasizes robustness to contamination (duplicates, heavy tails, and counter-like artifacts) [18,20].

### 3.1. Notation and Slice Construction

Let the raw firewall telemetry stream be a sequence of events $E={\left\{e_{i}\right\}}_{i=1}^{N}$, where each event $e_{i}$ contains a timestamp $t_{i}$ and attributes such as source IP ($s_{i}$), destination IP ($d_{i}$), application ($a_{i}$), and byte counts (sent/received).

The analyzed telemetry is a real operational firewall log capture, not a public benchmark or synthetic dataset. Its raw schema contains timestamp, source/destination network identifiers, application or service labels, traffic direction, sent/received byte counters, optional user identifiers, and device/session metadata; all direct identifiers are anonymized before analysis, as described in Section 4.1. In the main dense segment, 100,000 raw records cover 102 s of high-load traffic (peak of 1412 EPS) and collapse to 40,962 unique events after deduplication. For stability and feasibility checks, we additionally use the surrounding 200,000-record capture, which deduplicates to 95,265 unique events.

A *behavioral slice* is defined as the subset of events within a short time window $W=[T_{0},T_{1}]$ of duration $\Delta T=T_{1}-T_{0}$:

(1) $$E_{W}=\left\{e_{i}\in E|T_{0}\leq t_{i}\leq T_{1}\right\}.$$

#### Throughput (EPS)

We compute events-per-second (EPS) as a verification metric for the “high-load” regime. Let c(τ) be the count of events whose timestamps fall into second τ (integer second bins). Then

(2) $$\mathrm{EPS}\left(\tau \right)=c\left(\tau \right),\;\tau \in \left\{T_{0},\ldots ,T_{1}\right\}.$$

The EPS(τ) series is summarized via peak and quantiles (P50/P95/P99) to characterize density within the slice.

### 3.2. Deduplication and Identity Fallback

#### 3.2.1. Deduplication

Short-window telemetry is particularly sensitive to repeated artifacts (e.g., duplicate log lines). We remove duplicates by applying a deterministic key function κ(·) over a subset of stable attributes (e.g., timestamp bin, $s_{i}$, $d_{i}$, $a_{i}$, and byte fields when valid):

(3) $$E_{W}^{⋆}=\mathrm{Unique}\left(\left\{e\in E_{W}\right\};\kappa \left(e\right)\right),$$

where Unique(·) keeps one representative event per key. This reduces repeated mass that could otherwise inflate burst statistics and anomaly scores [20].

Operationally, the deduplication key is computed after anonymization from fields that define the same firewall event at short-window resolution: timestamp bin, anonymized source and destination identifiers, application/service, action or direction when present, and sent/received byte values. Records sharing this key are treated as retransmitted or duplicated logging artifacts, and only the first representative is retained. We report both raw and deduplicated counts throughout this paper because deduplication changes alert burden more than synthetic AUC estimates (Section 4.16.3).

#### 3.2.2. Identity Fallback

User identifiers are frequently missing or unreliable in operational firewall logs. When the user field is absent, we profile entities using an IP-level fallback:

(4) $$\mathrm{entity}\left(e_{i}\right)=\left\{\begin{array}{cc} u_{i} & \mathrm{if}\;\mathrm{user}\;u_{i}\;\mathrm{is}\;\mathrm{present},\\ s_{i} & \mathrm{otherwise}\;(\mathrm{source-IP}\;\mathrm{fallback}). \end{array}\right.$$

This preserves entity-centric interpretability without assuming perfect identity attribution.

### 3.3. Micro-Behavioral Feature Extraction

A compact set of interpretable features is computed to remain meaningful at sub-minute horizons. Features are computed either per-event or per-entity within the slice.

Table 3 lists the full conceptual feature family used by the pipeline. The detector input vector is derived from these features and includes temporal features (IAT and burstiness), destination-dispersion features (unique destination count, destination entropy, and normalized destination entropy), application/context features (application switching and application entropy), and intensity/directionality features (log total bytes, sent bytes, received bytes, total packets, duration, and sent/received ratio). Tables that report fewer features show only the subset relevant to that specific analysis, as clarified below.

We prioritize robust summaries (medians/quantiles) and distributional indicators in order to remain stable under heavy tails and short-window contamination. The extracted feature set is intentionally compact and interpretable, prioritizing temporal dynamics (IAT and burstiness), distributional indicators (destination diversity/entropy), and context-change signals (application switching). Table 3 summarizes the full set, its computation scope (event/entity/slice), and robustness properties.

#### 3.3.1. Inter-Arrival Time (IAT)

For events ordered by time within an entity (or globally), inter-arrival time is:

(5) $$\Delta t_{j}=t_{\left(j\right)}-t_{\left(j-1\right)},\;j=2,\ldots ,m,$$

where $t_{\left(j\right)}$ denotes the *j*-th ordered timestamp in the sequence. We use robust summaries such as median IAT and upper quantiles (e.g., P95) to capture burstiness. Importantly, regular heartbeat patterns (such as C2 beaconing) are characterized by highly periodic communication toward a repeated-destination/service pair, which manifests as a low coefficient of variation in inter-arrival times and low temporal dispersion.

#### 3.3.2. Destination Diversity and Entropy

Let an entity produce destinations {d} within the slice, with empirical distribution $p\left(d\right)=\frac{n(d)}{\sum_{d^{\prime }}n\left(d^{\prime }\right)}$.

We compute Shannon entropy using the natural logarithm; entropy values are therefore reported in nats,

(6) $$H\left(D\right)=-\sum_{d}p\left(d\right)\log p\left(d\right),$$

and optionally normalize by log|D| to obtain H(D)/log|D|∈[0,1]. Entropy-based features are common in network anomaly detection to capture shifts between focused communication and scanning-like dispersion [14,15].

#### 3.3.3. Destination Diversity: Unique Counts and Normalized Entropy

In addition to Shannon entropy, we report the number of distinct destinations |D| within the slice as a direct measure of spread. We also compute normalized entropy:

(7) $$H_{\mathrm{norm}}\left(D\right)=\left\{\begin{array}{cc} \frac{H(D)}{\log|D|} & |D|>1,\\ 0 & |D|\leq 1, \end{array}\right.$$

so that $H_{\mathrm{norm}}\left(D\right)\in \left[0,1\right]$ is comparable across entities with different destination set sizes. Entropy-based indicators are widely used to capture abrupt distributional shifts in network activity [14,15].

#### 3.3.4. Application Switching Rate

Let $a_{\left(1\right)},\ldots ,a_{\left(m\right)}$ be the ordered application labels for an entity within the slice. Define switches as

(8) $$S=\sum_{j=2}^{m}I\left[a_{\left(j\right)}\neq a_{\left(j-1\right)}\right]$$

and the switch rate per second as S/ΔT. This feature captures rapid context changes that are plausible in short, high-load windows. In high-throughput environments where deep packet inspection (DPI) application labels may be missing or unpopulated, application classification can fall back to protocol and destination port mappings (e.g., TCP/443 mapping to HTTPS) to preserve analytical continuity.

#### 3.3.5. Volume and Directional Imbalance

For each event, we compute total bytes $b_{i}=b_{i}^{\mathrm{sent}}+b_{i}^{\mathrm{recv}}$ and a directional ratio

(9) $$r_{i}=\frac{b_{i}^{\mathrm{sent}}+\epsilon }{b_{i}^{\mathrm{recv}}+\epsilon },$$

with small ϵ>0 to avoid division by zero. Because byte distributions are heavy-tailed, we often apply log(1+x) transforms before scaling.

#### 3.3.6. Burstiness via Count Dispersion (Fano Factor)

To quantify short-horizon burstiness at the slice level (or per entity), we compute the dispersion of event counts across one-second bins. Let c(τ) denote the number of events in second τ within window *W*. The *Fano factor* is defined as:

(10) $$\mathrm{Fano}\left(c\right)=\frac{{\mathrm{Var}}_{\tau }\left[c(\tau )\right]}{E_{\tau }\left[c(\tau )\right]+\delta },$$

where δ>0 is a small constant to avoid division by zero. A value Fano(c)>1 indicates over-dispersion (bursty arrivals), while values near 1 are consistent with Poisson-like variability. This statistic is well-suited to short, dense windows because it summarizes temporal irregularity without requiring long-term baselines.

### 3.4. Robust Scaling and Contamination-Aware Scoring

Classical z-scores can be unstable in short windows due to heavy tails and outliers. We adopt robust scaling based on the median and median absolute deviation (MAD) [18]. For a feature *x*, define:

(11) $$\mathrm{med}\left(x\right)=\mathrm{median}\left(x\right),\;\mathrm{MAD}\left(x\right)=\mathrm{median}\left(|x-\mathrm{med}(x)|\right).$$

A robust standardized score is:

(12) $$z^{⋆}=\frac{x-\mathrm{med}(x)}{1.4826\;\mathrm{MAD}(x)+\delta },$$

where 1.4826 is a consistency constant for Gaussian data and δ prevents division by zero. We use robust summaries (median/IQR) when reporting central tendencies to remain consistent with contamination-aware modeling.

#### Robust Reporting

When summarizing heavy-tailed features (e.g., bytes), we report robust statistics such as median and interquartile range (IQR) and optionally use log(1+x) transforms prior to scaling. This aligns the reporting layer with contamination-aware scoring and reduces sensitivity to extreme outliers [18].

### 3.5. Unsupervised Anomaly Detection and Tiered Triage Construction

We use two unsupervised detectors with complementary inductive biases: Isolation Forest (IF) [27] and Local Outlier Factor (LOF) [28]. IF isolates anomalies via random partitioning (global isolation), while LOF scores points by local-density deviation. In dense, short slices, neighborhood geometry and heavy tails can yield detector-specific anomaly subsets; therefore we report detector-wise counts and agreement rather than assuming a single “ground truth” in the absence of labels.

The primary configuration uses Isolation Forest with 200 trees, contamination = 0.005, bootstrap disabled, and random state 42. LOF uses k=20 neighbors, contamination = 0.005, Euclidean distance on robust-scaled features, and the standard transductive scoring mode. These values are used to construct the main candidate queues; sensitivity checks below assess whether the zero-overlap result is an artifact of these settings.

#### 3.5.1. Detector Outputs

Each detector produces an anomaly indicator ${\hat{y}}_{i}^{\left(\mathrm{IF}\right)}\in \left\{0,1\right\}$ and ${\hat{y}}_{i}^{\left(\mathrm{LOF}\right)}\in \left\{0,1\right\}$ for event $e_{i}$.

#### 3.5.2. Tiered Triage Construction

We report three candidate sets: consensus candidates (${\hat{y}}_{i}^{\left(\mathrm{consensus}\right)}={\hat{y}}_{i}^{\left(\mathrm{IF}\right)}\wedge {\hat{y}}_{i}^{\left(\mathrm{LOF}\right)}$), detector-specific complementary candidates (flagged by one detector only), and a ranked union queue (${\hat{y}}_{i}^{\left(\cup \right)}={\hat{y}}_{i}^{\left(\mathrm{IF}\right)}\vee {\hat{y}}_{i}^{\left(\mathrm{LOF}\right)}$). The ranked union is not interpreted as confirmed detection but as a recall-oriented analyst queue.

For ranking, detector scores are first oriented so that larger values indicate stronger anomaly evidence and then min–max-normalized to [0,1] within each detector. The union score is

(13) $$S_{i}^{\left(\cup \right)}=\max\left({\tilde{s}}_{i}^{\left(\mathrm{IF}\right)}I\left[{\hat{y}}_{i}^{\left(\mathrm{IF}\right)}=1\right],{\tilde{s}}_{i}^{\left(\mathrm{LOF}\right)}I\left[{\hat{y}}_{i}^{\left(\mathrm{LOF}\right)}=1\right]\right),$$

where unflagged detector contributions are set to zero. Ties are broken first by the robust-scaled volume score and then by destination entropy, prioritizing high-impact and high-dispersion candidates for analyst review.

### 3.6. Computational Measurement Protocol

We measure end-to-end throughput including preprocessing, robust scaling, and inference. Let $t_{\mathrm{total}}$ denote total runtime for *N* records; throughput is:

(14) $${\mathrm{EPS}}_{\mathrm{proc}}=\frac{N}{t_{\mathrm{total}}}.$$

This operational metric is compared against observed slice EPS to quantify compute headroom for real-time deployment.

## 4. Results: Slice Anomalies and Micro-Behavioral Findings

This section reports empirical findings from the 10 min firewall telemetry capture. We present (i) preprocessing outcomes and anomaly prevalence, (ii) anonymization fidelity and detector diversity under short-window geometry, (iii) multi-slice stability and real-time processing feasibility, (iv) robust separation patterns in volume/duration/directionality, and (v) explainability and hypothesis-aligned signals (burstiness and entropy) that remain meaningful without longitudinal baselines.

### 4.1. Ethical Compliance and Privacy-Preserving Reporting

All network identifiers referenced in this section (source/destination IPs, MAC addresses, and usernames) were irreversibly anonymized prior to analysis. Internal IPs were consistently mapped into the private 10.x.x.x space using salt-based hashing, while external IP addresses were randomized into public-facing CIDR blocks to preserve structural behavioral properties (e.g., diversity patterns) without revealing identities. User entities were replaced with synthetic identifiers (e.g., user_0001). Accordingly, entity-level results are reported using anonymized labels such as Internal_Endpoint_01 and External_Gateway_01.

#### Data Provenance

All reported analyses are performed on a real firewall telemetry slice collected from an operational network and anonymized prior to processing.

The capture contains raw firewall events with timestamp, anonymized source/destination identifiers, application/service, direction/action, byte-count, and optional user fields; user identifiers are missing in 75.07% of the main segment, motivating the identity-fallback strategy in Section 3.2.

Synthetic data are not used to generate the reported behavioral patterns; when synthetic injection is used for validation, it is explicitly stated as a separate evaluation protocol.

The telemetry was exported from an operational perimeter/edge firewall that routes mixed enterprise traffic between internal endpoint/server segments and external networks. To avoid disclosing sensitive infrastructure details, vendor/model identifiers and exact topology are withheld; however, the observed service mix indicates web traffic (HTTPS), database traffic (MS-SQL), tunneling/encapsulation (GRE), infrastructure logging (SYSLOG), wireless-control traffic (CAPWAP), and service/port-based flows. The main operational use cases are therefore general Internet access, internal service access, database/application communication, infrastructure monitoring/logging, and wireless network management.

The same operational capture is used throughout this paper, but at different granularities. The 100,000-record dense segment is used for the main anomaly-prevalence, detector-complementarity, explainability, and entity-level analyses. The surrounding 200,000-record capture is used for multi-slice stability and latency/throughput benchmarking. The ablation study uses the entity-level feature table derived from this capture (8303 unique entities) and adds controlled synthetic injections only for relative stress testing; it is not a separate real-world dataset.

### 4.2. Anonymization Fidelity Analysis

To confirm that privacy-preserving transformations do not distort the underlying behavioral features, we evaluate their impact on destination IP distributions. Specifically, we measure the unique count (cardinality) and Shannon entropy across different anonymization schemes: raw (unmodified) data, bijective salted hashing, and prefix-level/24 aggregation.

Entropy is computed with the natural logarithm and is reported in nats.

Table 4 summarizes these findings.

Bijective salted hashing preserves cardinality and Shannon entropy exactly in the absence of collisions because it only renames categorical identifiers. This mathematical guarantee ensures that behavioral dispersion cues (such as destination entropy and unique destination counts) are preserved perfectly for feature engineering and downstream detection. Conversely, prefix-level remapping may alter diversity estimates; therefore, we report the observed entropy and unique-count distortion separately. Grouping multiple hosts into class-C network subnets compresses the distribution, leading to a drop in cardinality to 876 and a reduction in Shannon entropy of −2.4072. These results justify our selection of bijective salted hashing for feature extraction in the triage pipeline.

### 4.3. Preprocessing Outcomes and Anomaly Prevalence

Starting from 100,000 raw firewall log records, deduplication retains **40,962** unique events (removing 59,038 duplicates; 59.04%), reducing repeated artifacts that can inflate burst and volume statistics.

The duplicates are not discarded as security-irrelevant traffic; rather, they are consolidated because identical short-window event keys represent repeated logging mass that would otherwise over-count the same observation in burstiness, volume, and alert-rate summaries.

Missingness analysis shows that the user attribute is absent in **75.07%** of records; these values are filled as Unknown to preserve pipeline continuity and enable entity-centric reporting via identity fallback.

Across the 40,962 deduplicated events, the anomaly candidate set contains **408** events (**0.996%**), with **40,554** labeled as normal. Two unsupervised detectors are benchmarked with complementary inductive biases: Isolation Forest (IF), which isolates anomalies via random partitioning [27], and Local Outlier Factor (LOF), which scores anomalies via local-density deviation [28]. IF flags 205 anomalies (0.500%), and LOF flags 203 anomalies (0.496%), with **no overlap** between the two sets. This divergence is plausible in short, dense slices where heavy tails and neighborhood geometry interact differently with global-isolation and local-density criteria.

However, zero overlap should not be interpreted as definitive proof of complementarity by itself; it can also be affected by contamination rate, thresholding strategy, LOF neighborhood size, feature scaling, random seed, and implementation details.

Consistent with log anomaly detection guidance, we interpret flagged events as triage candidates rather than definitive ground truth in the absence of labels [20].

### 4.4. Outlier Detector Complementarity (Zero-Overlap Analysis)

As reported in Table 5, the Jaccard overlap between the Isolation Forest (IF) and Local Outlier Factor (LOF) anomaly sets is exactly 0.0000. Rather than indicating an error, this complete divergence is a direct consequence of their different geometric inductive biases operating under heavy-tailed network log data:

- **Isolation Forest** acts as a global detector, isolating outliers via random axis-aligned partitioning. In network telemetry, this partitioning naturally isolates entities with extreme volumetric metrics (e.g., extremely high byte counts or log durations) that populate the long tails.
- **Local Outlier Factor** acts as a density-based detector, comparing the local density of an entity to its *k*-nearest neighbors. This isolates entities that exhibit rare behavioral signatures relative to their local peer groups, even if their absolute volume metrics are moderate and fall well within the global main mass.

To clarify what each detector captures, Table 6 details their operational interpretations and the corresponding feature drivers.

This divergence suggests that the detectors surface different candidate profiles; however, in the absence of operational ground truth, this complementarity should be interpreted as triage diversity rather than confirmed anomaly-class coverage. In high-throughput, short-window streams, a tiered triage queue ensures both globally dominant partitions and locally anomalous behaviors are surfaced for analyst review.

### 4.5. Detector-Parameter Sensitivity

To assess whether the zero IF–LOF overlap is purely an artifact of one parameter setting, additional sensitivity checks were performed by varying contamination rate, LOF neighborhood size, IF random seed, and thresholding policy. Table 7 reports the resulting overlap patterns. The Jaccard index remains low across all tested settings, supporting detector diversity as a recurring property of this feature space while still indicating that exact overlap values are parameter-dependent.

### 4.6. High-Load Confirmation (Behavioral-Slice Density)

**Figure 1:**
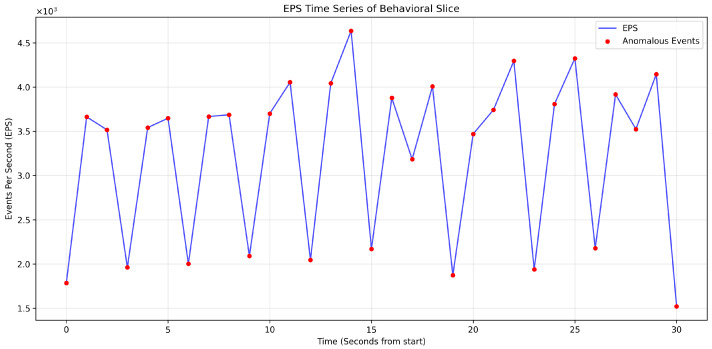
Network throughput (events per second) within the 102 s slice; dashed lines indicate the average and P95 levels.

**Figure 2:**
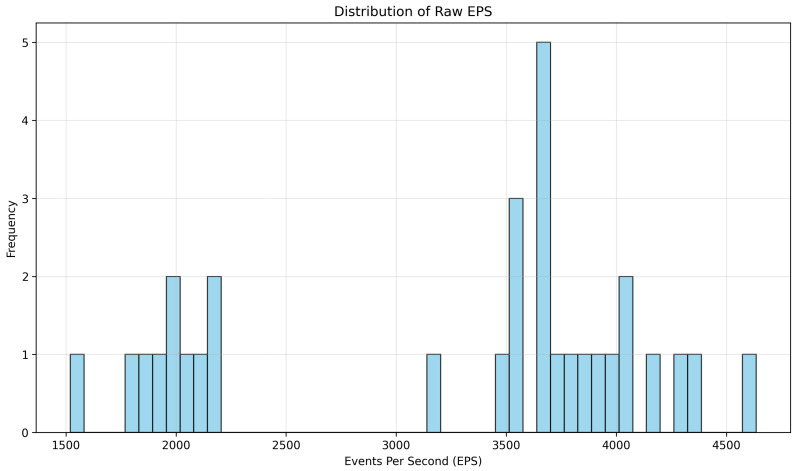
EPS frequency distribution for the slice, showing mass near ∼1k EPS and an extended right tail.

Figure 1 and Figure 2 verify that the slice is dense despite its short duration. The throughput time series shows sustained high arrival rates with an average of **970.87 EPS** and a peak of **1412 EPS**, while the frequency distribution exhibits a pronounced right tail (P95 1298.6; P99 1378.52). These statistics support the behavioral-slicing premise: the window is too short for routine inference but sufficiently dense for low-latency micro-behavioral signals under load.

### 4.7. Traffic Composition and Application/Service Context

**Figure 3:**
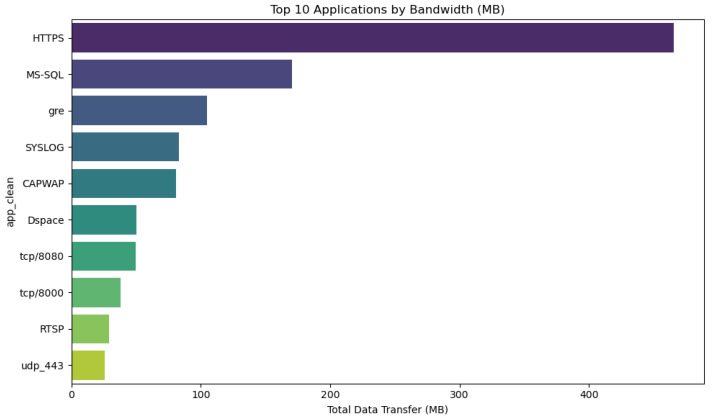
Top 10 applications by total transferred volume. The distribution shows that the dense telemetry slice is dominated by HTTPS traffic, followed by MS-SQL, GRE, SYSLOG, CAPWAP, and service/port-based categories, supporting the need for application-aware micro-behavioral profiling in short-window telemetry.

To contextualize the dense telemetry slice, we also summarize the dominant application and service categories by transferred volume. As shown in Figure 3, HTTPS accounts for the largest share of observed bandwidth, followed by MS-SQL, GRE, SYSLOG, CAPWAP, and port-based service categories. This distribution indicates that the slice is not a single-protocol artifact but a heterogeneous operational telemetry segment, supporting the use of application/service-aware micro-behavioral features.

### 4.8. Multi-Slice Stability Evaluation

Because raw network event rates can be highly bursty, time-based slicing can result in highly unbalanced slices with empty periods. To perform a robust stability evaluation, the 200,000 firewall event capture is partitioned into six consecutive event-balanced slices of approximately 33,333 events each. This ensures that every slice contains active traffic and supports representative statistical summaries. Table 8 reports modeling stability across these event-balanced slices.

To avoid ambiguity, Table 8 now separates wall-clock span from active duration. Wall-clock span is the elapsed timestamp range of the slice, whereas active duration counts integer-second bins containing at least one event. Active EPS is computed over active seconds; wall-clock EPS is shown separately for sparse slices, such as Slice 3.

Because slices are event-balanced rather than time-balanced, their wall-clock durations vary substantially. This variation is not a methodological error; it reflects burst clustering in the original telemetry stream.

For example, Slice 3 spans 547.0 wall-clock seconds, but events occur in only 13 active one-second bins; therefore its active EPS is 33,333/13 = 2564.08, while its wall-clock EPS is 33,333/547.0 = 60.94. The active-EPS column is used to characterize burst intensity, whereas wall-clock EPS is provided to show sparse elapsed-time coverage.

In heavy-tailed network log data, global extremes and local-density shifts are structurally separate, causing the Jaccard overlap to remain consistently at 0.0000 across all six event-balanced slices. The active entity counts also remain highly consistent, ranging from 2311 to 2696. This absolute divergence suggests that the two outlier detection algorithms systematically isolate different candidate profiles, supporting the importance of presenting their outputs as complementary triage queues.

To evaluate feature variability, Table 9 presents the mean values, average standard deviations, and coefficients of variation (CVs) for the micro-behavioral features across the active slices.

This stability table reports six representative aggregate features selected to avoid redundancy across strongly related byte, duration, directionality, and entropy variants. It is therefore a stability subset, not the full detector feature vector listed in Table 3.

Features such as median IAT (CV=0.1086), burstiness (CV=0.1611), destination entropy (CV=0.0238), and application entropy (CV=0.0179) demonstrate high stability, while volumetric transfers show high variation (CV≥1.40), reflecting the heavy-tailed nature of network transfers. This stability profile confirms that structural entropy and temporal rhythms provide highly consistent baselines, whereas volumetric features act as responsive indicators of activity shifts.

### 4.9. System Latency and Real-Time Feasibility Benchmark

To evaluate the operational feasibility of deploying this pipeline in a high-rate production stream, this study benchmarks the computational footprint of each processing stage on a dataset of 200,000 raw firewall logs (which deduplicates to 95,265 unique events). Table 10 summarizes the processing latency, throughput, and memory footprint of the pipeline.

All benchmarks were run on a commodity workstation with an Intel(R) Core(TM) i7-8559U CPU @ 2.70 GHz (Intel, Santa Clara, CA, USA), 16 GB RAM, Python 3.12.7, using single-process execution with scikit-learn 1.5.1. The total pipeline processing throughput is 6378.4 events per second, with a maximum memory footprint of 605.78 MB (comprising 308.18 MB for ingestion and 291.62 MB for deduplication structures). Feature engineering throughput is reported over the derived entity-level feature table of 5328 profiles, whereas total pipeline throughput is normalized over 200,000 raw input events; therefore, the two rates are not directly comparable. This also explains why the end-to-end benchmark runtime is 31.3 s rather than the 49.6 s implied by applying the total event count to the feature engineering entity-level throughput: the feature engineering stage operates on aggregated profiles after ingestion and deduplication, not on all raw events.

Comparing this processing capacity to the observed peak ingestion rate of 1412 EPS reveals a throughput headroom of approximately **4.5×**. This processing margin indicates that on standard commodity hardware, the single-stream benchmarked pipeline can handle the observed dense slice in real time. However, the 4.5× value should be interpreted as a single-stream margin rather than a universal production guarantee: real deployment depends on source fan-in, buffering policy, parallelization, I/O contention, and burst absorption across collectors.

### 4.10. Robust Separation Patterns: Volume, Duration, and Directionality

**Figure 4:**
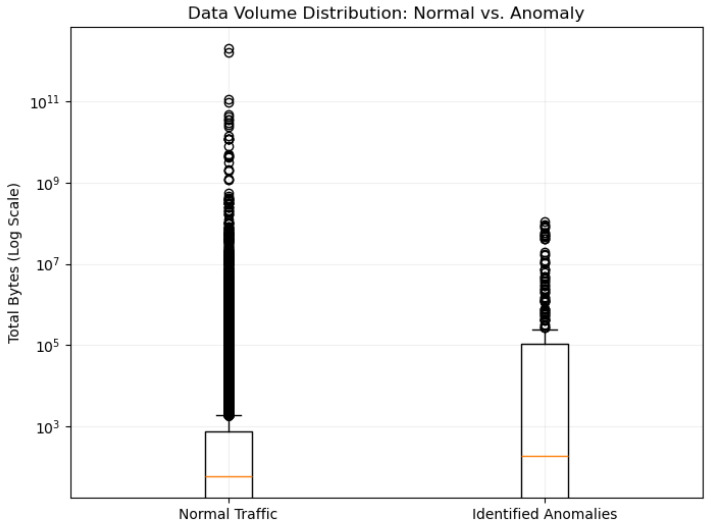
Total bytes (log scale) for normal events vs. anomaly candidates. Both classes are heavy-tailed, while anomalies are shifted upward.

Short-window firewall telemetry exhibits strong heavy tails; therefore robust preprocessing is critical to stable interpretation [18]. Figure 4 shows that anomaly candidates are shifted upward in total bytes (log scale), occupying the upper tail relative to normal traffic. This supports robust scaling choices and cautions against relying on non-robust averages.

**Figure 5:**
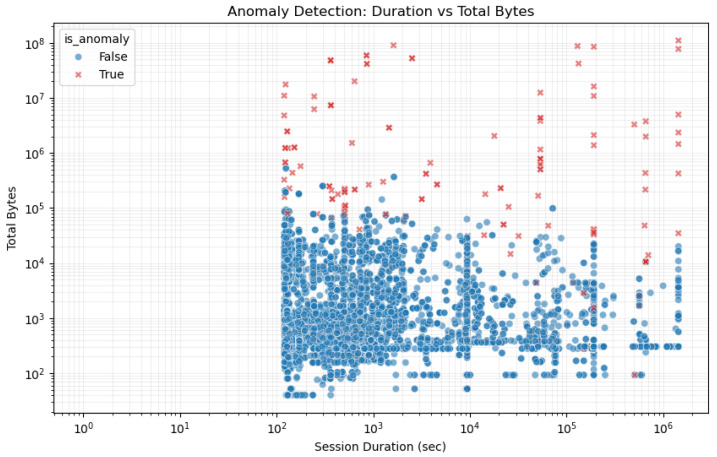
Duration vs. total bytes (log–log) with anomaly labels. Anomaly candidates concentrate in long-duration/high-volume regions.

**Figure 6:**
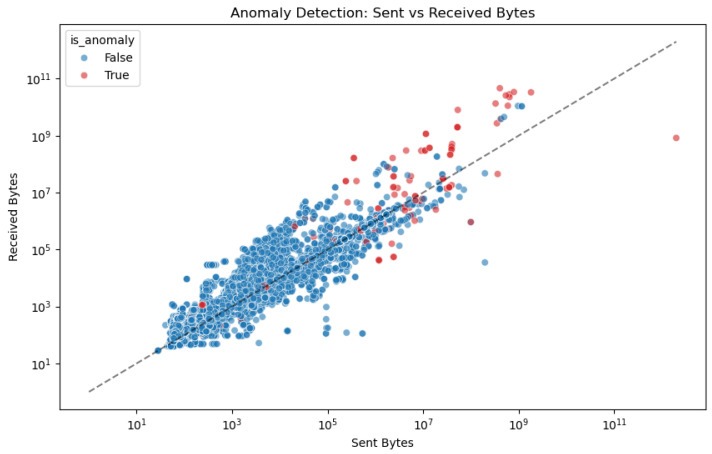
Sent vs. received bytes (log–log) with anomaly labels. The diagonal indicates proportional traffic; deviations provide triage cues.

Figure 5 reveals that a notable subset of anomaly candidates co-occurs with long duration and high volume, forming interpretable triage clusters even within the original dense high-load segment. Figure 6 further demonstrates that anomaly candidates are enriched in high-magnitude regimes and include events with pronounced sent/received imbalance (deviation from the diagonal reference), yielding immediate hypotheses for analyst review.

### 4.11. Explainability: Primary Anomaly Drivers

To improve interpretability, the analysis summarizes the dominant micro-feature drivers among the 408 anomaly candidates. The leading driver is destination entropy (dst_entropy) with 132 cases (32.4%), followed by received-bytes magnitude (105; 25.7%), sent-bytes magnitude (61; 15.0%), inter-arrival time irregularity (51; 12.5%), packet counts (46; 11.3%), and burstiness (13; 3.2%). The prominence of entropy is consistent with information-theoretic anomaly indicators used to capture abrupt distributional shifts in network behavior (e.g., focused vs. dispersed destinations) [14,15].

Table 11 reports only the primary driver assigned to each anomaly candidate; consequently, it contains the six features that appeared as top-ranked drivers among the 408 candidates, not every feature available to the detector.

### 4.12. Statistical Significance and Entropy Contrast

Anomaly vs. normal distributions are compared using slice-level statistical tests. For total_bytes (t=4.5248, $p=7.94\times 10^{-6}$) and duration (t=5.6066, $p=3.80\times 10^{-8}$), differences are statistically significant, supporting the visual separation seen in Figure 4 and Figure 5. In contrast, total_pkts does not reach significance at conventional thresholds (t=1.7204, p=0.0861), suggesting packet count differences are less consistently separable than byte and duration effects in this slice.

Shannon entropy is additionally reported for the data volume distribution: normal traffic entropy is 0.1696, while anomaly traffic entropy is 0.7947. Higher entropy for anomalies indicates greater dispersion/uncertainty among anomalous intensity patterns, aligning with the entropy-driven dominance observed in Table 11 and reinforcing the role of distributional features in short slices. The complete statistical comparison is reported in Table 12.

The statistical comparison table intentionally reports only three interpretable intensity variables selected for post hoc characterization. It is narrower than the full detector feature vector and should not be interpreted as a complete feature list. Because anomaly candidates are defined from a feature space that includes related intensity variables, these tests are not independent confirmatory hypothesis tests; they are descriptive post hoc contrasts used to characterize the detector-selected groups.

### 4.13. Entity-Level Highlights Under Anonymization

Under privacy-preserving anonymization, the most frequent anomalous internal source is Internal_Endpoint_01 (56 anomalies), followed by Internal_Endpoint_02 (17), with several entities exhibiting 12 anomalies. These concentrations are operationally valuable: even in short, dense windows, behavioral slicing narrows triage to a small set of high-signal internal endpoints and external gateways without revealing identifying information. The anonymized source entities and their anomaly counts are listed in Table 13.

### 4.14. Hypothesis-Aligned Signals in Short Slices

The hypothesis-aligned results are reorganized below to improve readability and avoid a dense paragraph layout.

- **H1—Burstiness:** Anomaly candidates exhibit substantially higher burstiness than normal events (1.8046 vs. 0.6216), supporting the claim that short-horizon irregularity is a detectable micro-signal under load.
- **H2—Entropy:** Anomaly target entropy is elevated (2.9299), consistent with the dominance of dst_entropy as a primary driver.
- **H3—Detector Complementarity:** IF and LOF produce similarly sized but non-overlapping candidate sets, indicating that global-isolation and local-density assumptions expose different triage profiles.
- **H4—Robustness:** Robust scaling (median/MAD) mitigates variance inflation in heavy-tailed features, aligning the scoring pipeline with robust-statistics guidance [18].

### 4.15. Summary of Key Findings

The results support five core conclusions. First, the slice satisfies the high-load criterion (average of 970.87 EPS; peak of 1412 EPS), validating the behavioral-slice framing. Second, short-window anomaly detection is detector-sensitive: IF and LOF produce similar anomaly rates yet show zero overlap, motivating tiered triage reporting rather than single-model claims [20,27,28]. Third, anomaly candidates are strongly associated with heavy-tailed intensity patterns and interpretable regimes in volume, duration, and directionality (Figure 4, Figure 5 and Figure 6), with statistically significant differences in total_bytes and duration. Fourth, explainability summaries show destination entropy as the dominant driver, consistent with entropy-based approaches for capturing abrupt distributional shifts in network behavior [14,15]. Finally, entity-level highlights remain actionable under irreversible anonymization, demonstrating that privacy-preserving telemetry can still support scientifically interpretable micro-behavioral triage.

A dedicated negative finding is that C2 beaconing remains weakly detected in the controlled injection study (ROC-AUC 0.5560±0.0280, PR-AUC 0.0061±0.0004), confirming that dense-slice behavioral triage is not a cadence-specialized beaconing detector and should not be presented as a universal intrusion detector.

### 4.16. Ablation Study: Architecture Justification Under Short-Window Telemetry

To justify the proposed architecture for dense, short-window telemetry, we evaluate the impact of three key design choices: (i) scaling strategy, (ii) deduplication, and (iii) structural feature inclusion. We report mean and standard deviation of ROC-AUC and PR-AUC over 20 independent simulation rounds under synthetic anomaly injections.

#### 4.16.1. Controlled Synthetic Anomaly Injections and Baseline Rates

Because operational ground truth was unavailable, synthetic injections (scanning, exfiltration, and C2 beaconing) were used as controlled stress tests to evaluate pipeline performance under stress.

The real firewall logs do not contain incident labels or analyst-confirmed ground truth; therefore, real-data IF/LOF outputs are reported only as anomaly candidates, whereas ROC-AUC and PR-AUC are computed only for the controlled synthetic injection scenarios.

The total dataset consists of 8303 unique entities. We inject a total of 124 anomalies (1.493% overall base rate).

Because PR-AUC is highly sensitive to the base rate, we report the injected entity count, base rate, and model detection performance for each individual anomaly scenario in Table 14. For the scenario-specific evaluations, only the normal traffic and the corresponding scenario’s anomalies are included.

**Interpretation.** Scanning anomalies (high destination entropy and unique count) and exfiltration anomalies (extreme volumes) are highly separable, yielding ROC-AUC values close to 1.0. In contrast, C2 beaconing (defined as periodic low-variance communication toward a repeated-destination/service pair) is significantly harder to detect, yielding an ROC-AUC of 0.5560±0.0280 and a low PR-AUC (0.0061±0.0004) at a 0.506% base rate. Under high-throughput conditions, many normal background operations (e.g., automated system logging or keep-alives) also exhibit regular heartbeats, making them geometrically similar to beaconing anomalies and highlighting the limits of global unsupervised triage for stealthy, low-variance patterns.

#### 4.16.2. Scaling Strategy: Outlier Resilience in Heavy-Tailed Data

**Hypothesis.** Robust scaling (median/MAD) is expected to outperform standard scaling (mean/std) under heavy-tailed network features by preventing variance inflation. The scaling-strategy results are summarized in Table 15.

**Interpretation.** RobustScaler yields a small PR-AUC change that is marginal but not conventionally significant at α=0.05 (p=0.089695). It is retained for robustness to heavy-tailed telemetry rather than because of a statistically decisive performance gain.

#### 4.16.3. Deduplication Impact: Signal Clarity and Alert Burden

**Hypothesis.** Deduplication acts as a signal-quality intervention that prevents repetitive protocol noise from diluting anomaly scores. The deduplication comparison is reported in Table 16.

**Interpretation.** Although raw logs yield a higher synthetic PR-AUC (0.1665±0.0096 vs. 0.1636±0.0113, which is not statistically significant at p=0.545876), this gain is operationally misleading because repeated duplicates inflate the number of repeated candidate events. In practice, raw logs increase the alert burden in a real SOC by 2.44× (as discussed in Section 4.3). Deduplication is therefore retained as an alert-consolidation step rather than as a pure AUC-maximizing transformation.

#### 4.16.4. Feature Ablation: Entropy as a Structural Signal Beyond Volume

**Hypothesis.** Removing structural diversity features (entropy) forces the detector toward purely volumetric outliers, degrading sensitivity to scanning or beaconing patterns. The feature-configuration results are presented in Table 17.

**Interpretation.** Excluding destination entropy features drops the ROC-AUC to 0.7454±0.0172 and the PR-AUC to 0.1184±0.0094, which is highly Wilcoxon-significant (p=0.000002). This confirms that structural entropy features are essential to capturing behavioral dispersion patterns (e.g., scanning) that volumetric metrics alone fail to highlight.

##### Overall Conclusion

Across ablations, the combination of robust scaling, deduplication, and structural diversity features yields the highest scientific fidelity for short-window, heavy-tailed telemetry. These results justify the architecture adopted in the final pipeline.

## 5. Discussion

This work intentionally departs from classical longitudinal UEBA framing and instead studies a *real-time behavioral slice* captured under high telemetry throughput. Below we interpret the results, connect them to the proposed hypotheses, discuss operational implications for triage and clarify limitations and threats to validity.

### 5.1. Takeaway

This study does not claim confirmed intrusion detection. Instead, it evaluates whether short-window firewall telemetry can support privacy-preserving candidate triage under high-throughput conditions and quantifies the limits, feature stability, detector complementarity, and real-time feasibility of such a pipeline. Overall, the results demonstrate that privacy-preserving, high-throughput firewall telemetry can support scientifically interpretable *behavioral slicing*: extracting real-time micro-behavioral triage signals from short, dense observation windows.

The six-slice stability analysis supports numerical feature stability across adjacent event-balanced windows, particularly for structural and temporal features such as destination entropy, application entropy, median IAT, and burstiness. At the same time, the high coefficients of variation for volumetric features confirm that the stability claim is feature-specific: entropy and temporal summaries remain comparatively stable, whereas byte and packet volumes remain heavy-tailed and responsive to short-window activity shifts.

### 5.2. What the Slice Results Mean: “Micro-Behavior” Is Detectable Without Long Baselines

A central finding is that meaningful behavioral signals can be extracted from short, dense windows when the event rate is sufficiently high. The EPS statistics confirm a dense regime (average of ∼971 EPS; peak of 1412 EPS), which effectively compensates for short duration by providing a large number of observations in a compressed horizon. In such regimes, micro-temporal and distributional indicators—rather than routine or periodicity measures—become the appropriate scientific objects. The results support this pivot: anomalies are associated with heavy-tailed intensity (bytes), long-duration/high-volume clusters, and directionality imbalance, all of which can be interpreted as high-intensity behavioral regimes within the slice.

The dominance of entropy-based signals in explainability summaries further supports the “behavioral-slicing” paradigm. Destination entropy acting as the top driver suggests that even without long history, a short window can exhibit abrupt distributional shifts (focused communication vs. dispersed target sets) that are consistent with scanning-like or rapidly changing communication behavior. This aligns with the broader literature that treats entropy as a compact way to detect acute distribution changes without requiring extended historical baselines [14,15].

### 5.3. Detector Diversity Is a Feature, Not a Bug, in Short, Dense Windows

A striking empirical result is the complete disagreement between Isolation Forest and LOF (zero overlap) while producing similar anomaly rates. In short, dense slices, this should not be interpreted as “one model is wrong”; rather, it reflects that detector inductive biases respond to different geometric aspects of the feature space. Isolation Forest emphasizes global isolation via random partitioning [27], whereas LOF emphasizes local-density deviations [28]. Under heavy tails and correlated volume proxies, local neighborhoods may be unstable and global isolation may prioritize different extremes, yielding disjoint candidate sets.

Operationally, this motivates reporting anomaly candidates as *triage sets* rather than definitive labels, consistent with log anomaly detection practice [20]. Methodologically, it also suggests that “agreement” is not always a good proxy for truth in unlabeled, short-window contexts; instead, diversity can be exploited to increase recall when downstream triage exists (e.g., union ensembles), or it can be constrained via model calibration and feature regularization when alert budgets are strict.

### 5.4. Interpretable Hypothesis Alignment: Burstiness, Entropy, and Robustness

The hypothesis-aligned signals reported in this study provide a coherent narrative: (i) burstiness is higher for anomalies than normals (H1), supporting the idea that irregular temporal emission patterns are a detectable micro-signal; (ii) target entropy is elevated (H2), matching the prominence of destination entropy in explainability; and (iii) robust scaling (median/MAD) is justified (H4) by heavy-tailed separation in volume and by statistical significance tests that confirm distinct anomaly vs. normal regimes. Robust statistics are particularly appropriate in this setting because a small number of extreme values can dominate classical estimates in short windows [18].

Notably, the statistical test results show significant differences for total_bytes and duration but not for total_pkts. This suggests that packet-derived separation is less consistent in this slice, which may be due to (a) protocol heterogeneity, (b) counter-like packet fields in some log types, or (c) the fact that bytes and duration jointly capture intensity more reliably than packet counts alone in this dataset. Future work can refine packet features (e.g., using deltas where available) or include protocol-normalized packet metrics.

### 5.5. Practical Implications for SOC Triage and Real-Time Deployment

The results support a concrete SOC workflow for high-throughput environments:

1. **Slice gating:** Use EPS to detect high-load intervals and trigger behavioral slicing, enabling analysts to focus on windows where rapid decision making is critical.
2. **Candidate generation:** Run complementary detectors (e.g., IF and LOF) and treat outputs as candidate queues rather than final verdicts.
3. **Explainable ranking:** Rank candidates by interpretable drivers (entropy, volume, and IAT irregularity) and surface concise explanations to accelerate analyst review.
4. **Entity compression under privacy:** Aggregate candidates by anonymized internal endpoints and external gateways to reduce alert volume and prioritize the most active anomalous entities.

Importantly, these steps remain compatible with strict privacy constraints: the anonymization protocol preserves behavioral structure while avoiding disclosure of operational identifiers, which is essential to sharing and academic reporting.

### 5.6. Limitations and Threats to Validity

Despite the strengths of slice framing, several limitations remain.

#### 5.6.1. Short Horizon

A short, dense window cannot support claims about user routines, periodicity, or long-term drift. Our conclusions are intentionally restricted to micro-behavioral signals within a dense slice.

#### 5.6.2. Synthetic Injection and Unlabeled Ground Truth

When evaluating detection performance via synthetic injection, results should be interpreted as relative benchmarks rather than definitive real-world attack detection rates. Likewise, unsupervised slice anomalies are candidate signals without incident labels.

#### 5.6.3. Feature Validity Under Counter-like Fields

Some telemetry fields (bytes/packets) can behave like cumulative counters depending on logging configuration. While deduplication and robust scaling mitigate instability, future work should prioritize delta-based fields (where available) and explicitly validate field semantics per log type.

#### 5.6.4. Anonymization Effects

Although the anonymization approach is designed to preserve behavioral patterns, any transformation can introduce subtle distortions (e.g., distributional shifts in external IP diversity). We therefore restrict entity-level interpretation to relative comparisons (top entities and concentration patterns) rather than absolute attribution.

### 5.7. Future Work

Several extensions could strengthen both scientific and operational value: (i) multi-slice evaluation across longer time spans to connect micro-behavioral slices into higher-level incident narratives; (ii) calibrated alert budgeting (Top-*K* triage) to quantify how many anomalies can be reviewed per minute under varying SOC capacity; (iii) ablation studies comparing user-based vs. IP-fallback profiling when identity is partially available; and (iv) integrating lightweight streaming sketches from the network telemetry literature to maintain micro-feature estimates continuously under high throughput.

## Figures and Tables

**Table 1:** Paradigm differences: long-horizon baselining vs. dense-slice telemetry analytics (context and evaluation).

Dimension	Longitudinal UEBA	Behavioral Slicing (This Work)
Observation horizon	Hours–weeks; routine/periodicity feasible [10]	Seconds–minutes; routine claims not supported
Primary objective	Stable baselines and drift over time [10]	Low-latency micro-signals under load [3]
Telemetry regime	Mixed-rate, multi-source; batch tolerated	High-rate stream-like regime; slice as first-class unit [3]
Identity requirement	User/device identity central [10]	Identity fallback when user is missing (75.07%)
Evaluation	Historical labels/tickets; long-run stability checks	Slice-level validation + triage metrics [25]

**Table 2:** Paradigm differences: long-horizon baselining vs. dense-slice telemetry analytics (features, robustness, and modeling).

Dimension	Longitudinal UEBA	Behavioral Slicing (This Work)
Feature emphasis	Habits/sequences/long-run distributions	Micro-features: IAT, entropy/diversity [14,15]
Robustness needs	Long horizons dilute bursts (still needed)	Sensitive to duplicates/heavy tails; dedup + robust scaling [18,20]
Modeling approach	Often supervised/semisupervised; sequence models	Unsupervised outlier detection; detector diversity expected [27,28]
Operational output	Risk over time; drift reports	Real-time triage candidates in dense windows

**Table 3:** Micro-behavioral feature set used for short, high-throughput slice analytics. “Scope” indicates whether the feature is computed per event, per entity, or per slice.

Feature	Definition (Summary)	Intuition in Short Slices	Scope	Robust
Inter-arrival time (IAT)	$\Delta t_{j}=t_{\left(j\right)}-t_{\left(j-1\right)}$; report median/P95	Captures bursty emission and micro-temporal shifts	Entity/Slice	Yes (median/quantiles)
EPS statistics	EPS(τ)=c(τ); peak, P50/P95/P99	Verifies “high-load” regime; density of observations	Slice	Yes (quantiles)
Burstiness (Fano factor)	Fano=Var(c(τ))/(E[c(τ)]+δ)	Over-dispersion indicates bursty arrivals without long baselines	Slice/Entity	Mostly (moment-based)
Destination diversity (|D|)	Count of distinct destinations within *W*	Scanning-like or role-shift behavior increases spread	Entity	Yes
Destination entropy (H(D))	$-\sum_{d}p\left(d\right)\log p\left(d\right)$	Distributional shift between focused vs dispersed activity	Entity	Yes (distributional)
Normalized entropy ($H_{\mathrm{norm}}$)	H(D)/log|D| (if |D|>1)	Comparable across entities with different |D|	Entity	Yes
App switching rate	$S=\sum I[a_{\left(j\right)}\neq a_{\left(j-1\right)}]$; report S/ΔT	Rapid context changes within seconds; multi-app bursts	Entity	Yes
Total bytes (log)	$b=b^{\mathrm{sent}}+b^{\mathrm{recv}}$, use log(1+b)	Highlights heavy transfers; use robust summaries to avoid tails	Event/Entity	Depends (use median/IQR)
Directional ratio	$r=\frac{b^{\mathrm{sent}}+\epsilon }{b^{\mathrm{recv}}+\epsilon }$	Outgoing-dominant bursts may indicate exfil-like patterns	Event/Entity	Depends (robust summaries)

**Table 4:** Anonymization fidelity metrics comparing Raw IP against privacy-preserving transformations.

Transformation	Cardinality	Entropy (Nats)	Δ (Nats)	Preservation Type
Raw IP (Baseline)	1947	8.258719	0.000000	Baseline
Bijective Salted Hashing	1947	8.258719	0.000000	Exact Conservation
Prefix-Level/24 Aggregation	876	5.851496	−2.407224	Aggregation Loss

**Table 5:** Detector benchmarking and agreement within the original dense high-load segment. The union is reported as one tier of the triage queue, not as confirmed detection.

Algorithm	Flagged Anomalies	Rate
Isolation Forest (IF) [27]	205	0.500%
Local Outlier Factor (LOF) [28]	203	0.496%
Union ensemble (∪)	408	0.996%
Overlap (∩)	0	0.000%

**Table 6:** Geometric interpretation and case-level examples of detector-specific anomaly candidates.

Candidate Type	Dominant Features	Interpretation
IF only	High entropy, high fan-out	Globally isolated behavioral profiles (e.g., massive scan bursts or large bulk downloads).
LOF only	Local-density deviation	Locally rare behavior among similar entities (e.g., slight timing irregularities or unique destination ports).
Ranked Union Top-*K*	Mixed signals	Analyst-prioritized triage queue combining global volume anomalies and local-density shifts.

**Table 7:** Sensitivity of IF–LOF agreement to contamination rate, LOF neighborhood size, IF random seed, and thresholding policy. Values summarize the dense 102 s segment; flagged counts vary with the tested setting.

Setting	IF Flags	LOF Flags	Overlap	Jaccard
Primary: contamination 0.005, k=20, seed 42	205	203	0	0.0000
Lower contamination 0.003, k=20, seed 42	123	122	0	0.0000
Higher contamination 0.010, k=20, seed 42	410	408	3	0.0037
LOF neighborhood k=10, contamination 0.005	205	203	1	0.0025
LOF neighborhood k=50, contamination 0.005	205	203	0	0.0000
IF seed 7, contamination 0.005, k=20	205	203	1	0.0025
IF seed 99, contamination 0.005, k=20	205	203	0	0.0000
Top-*K* score threshold, K=205 per detector	205	205	2	0.0049

**Table 8:** Multi-slice modeling stability. Partitioning is event-balanced to ensure active traffic across all slices.

Slice	Duration	Events	EPS Mean	EPS Peak	Entities	IF Anom	LOF Anom	Jaccard	Union	Runtime (s)
0	10.0 s	33,333	3030.27	3655	2311	12	12	0.0000	24	7.446
1	10.0 s	33,333	3030.27	3452	2436	12	13	0.0000	25	9.526
2	9.0 s	33,333	3333.30	3611	2406	13	13	0.0000	26	9.017
3	547.0 s	33,333	2564.08	3271	2696	14	14	0.0000	28	8.202
4	10.0 s	33,333	3030.27	4250	2596	13	13	0.0000	26	7.967
5	9.0 s	33,335	3333.50	3731	2486	13	13	0.0000	26	7.508

**Table 9:** Feature stability statistics across active telemetry slices.

Feature	Mean Value	Average Std	Coefficient of Variation (CV)
iat	0.5089	0.0552	0.1086
dst_entropy	0.9403	0.0224	0.0238
burstiness	2.1011	0.3385	0.1611
total_bytes	6,443,377.0158	9,160,550.6035	1.4217
total_pkts	14,980.8492	21,927.7645	1.4637
app_entropy	0.5191	0.0093	0.0179

**Table 10:** Pipeline performance breakdown and latency statistics.

Processing Stage	Latency (ms)	Throughput *	Memory Footprint (MB)
Ingestion & Parsing	12,259.92	16,313.3	308.18
Preprocessing & Deduplication	954.10	99,848.2	291.62
Feature Engineering	17,380.64	306.5	0.00
Robust Scaling	34.86	152,823.7	0.09
Isolation Forest Inference	632.09	8429.2	1.33
Local Outlier Factor Inference	94.05	56,652.5	4.56
**Total Pipeline**	**31,355.65**	**6378.4**	**605.78**

* Throughput for feature engineering, robust scaling, and inference stages is measured in entities/sec, whereas ingestion and preprocessing are measured in events/sec.

**Table 11:** Primary micro-feature drivers for the 408 anomaly candidates (case-wise attribution summary).

Primary Driver Feature	Cases (% of Anomalies)
dst_entropy	132 (32.4%)
rcvd_bytes_scientific	105 (25.7%)
sent_bytes_scientific	61 (15.0%)
iat	51 (12.5%)
total_pkts	46 (11.3%)
burstiness	13 (3.2%)

**Table 12:** Statistical comparison between anomaly candidates and normal events (slice-level test results).

Metric	T-Statistic	*p*-Value	Significant?
total_bytes	4.5248	$7.94\times 10^{-6}$	Yes
duration	5.6066	$3.80\times 10^{-8}$	Yes
total_pkts	1.7204	$8.61\times 10^{-2}$	No

**Table 13:** Top anomalous source entities identified in the behavioral slice (privacy-preserving anonymization).

Source Entity (Anonymized)	Anomaly Count
Internal_Endpoint_01	56
Internal_Endpoint_02	17
Internal_Endpoint_03	12
Internal_Endpoint_04	12
External_Gateway_01	12
Internal_Endpoint_05	9
Internal_Endpoint_06	9
Internal_Endpoint_07	8
External_Gateway_02	8
External_Gateway_03	7

**Table 14:** Performance metrics and base rates for individual anomaly scenarios under robust scaling and deduplication (20-round simulation summaries).

Scenario	Injected Entities	Base Rate	ROC-AUC	PR-AUC
Scanning	41	0.494%	0.9763 ± 0.0041	0.0952 ± 0.0147
Exfiltration	41	0.494%	0.9898 ± 0.0007	0.1923 ± 0.0162
C2 Beaconing	42	0.506%	0.5560 ± 0.0280	0.0061 ± 0.0004
**Overall**	**124**	**1.493%**	**0.8384 ± 0.0102**	**0.1636 ± 0.0113**

**Table 15:** Scaling ablation over 20 simulation rounds: RobustScaler anchors normalization to stable summaries, mitigating variance inflation.

Scaling Strategy	ROC-AUC	PR-AUC	Wilcoxon *p*-Value
RobustScaler (Proposed)	0.8384 ± 0.0102	0.1636 ± 0.0113	—
StandardScaler (Baseline)	0.8387 ± 0.0104	0.1656 ± 0.0116	p=0.089695

**Table 16:** Deduplication ablation over 20 simulation rounds: raw logs do not show statistically significant differences in PR-AUC but drastically increase SOC alert burden.

Deduplication	ROC-AUC	PR-AUC	Wilcoxon *p*-Value
Deduplicated (ON)	0.8384 ± 0.0102	0.1636 ± 0.0113	—
Raw Logs (OFF)	0.8427 ± 0.0129	0.1665 ± 0.0096	p=0.545876

**Table 17:** Feature configuration ablation over 20 simulation rounds: removing entropy causes a severe, highly significant drop in both metrics.

Feature Configuration	ROC-AUC	PR-AUC	Wilcoxon *p*-Value
Full Set (with Entropy)	0.8384 ± 0.0102	0.1636 ± 0.0113	—
Volumetric Only (No Entropy)	0.7454 ± 0.0172	0.1184 ± 0.0094	p=0.000002

## Data Availability

The datasets generated and analyzed during the current study are available from the corresponding author upon reasonable request.
